# DED or alive: assembly and regulation of the death effector domain complexes

**DOI:** 10.1038/cddis.2015.213

**Published:** 2015-08-27

**Authors:** J S Riley, A Malik, C Holohan, D B Longley

**Affiliations:** 1 Drug Resistance Group, Centre for Cancer Research & Cell Biology, Queen's University Belfast, Belfast, UK

## Abstract

Death effector domains (DEDs) are protein–protein interaction domains initially identified in proteins such as FADD, FLIP and caspase-8 involved in regulating apoptosis. Subsequently, these proteins have been shown to have important roles in regulating other forms of cell death, including necroptosis, and in regulating other important cellular processes, including autophagy and inflammation. Moreover, these proteins also have prominent roles in innate and adaptive immunity and during embryonic development. In this article, we review the various roles of DED-containing proteins and discuss recent developments in our understanding of DED complex formation and regulation. We also briefly discuss opportunities to therapeutically target DED complex formation in diseases such as cancer.

## Facts

- FADD, FLIP, procaspase-8 and procaspase-10 all contain death effector domains (DEDs).
- The DED is a conserved protein sub-domain that mediates important protein–protein interactions.
- DED-containing proteins form a variety of complexes that regulate key cellular processes, most notably apoptosis, necroptosis and autophagy.
- Recent reports also highlight the critical role of DED proteins in other key processes linked to development and inflammation.

## Open Questions

- Does caspase-10 (absent in mice) have overlapping functions with caspase-8, or is it functionally distinct?
- Under what physiologically relevant conditions does necroptosis occur rather than apoptosis?
- In which cellular contexts are FADD, FLIP, (pro)caspase-8 and (pro)caspase-10 critical for regulating autophagy?
- What are the best ways of targeting DED-containing proteins to therapeutically activate cell death (e.g., in cancers) or prevent cell death (e.g., in neurodegenerative diseases)?
- Are DED-containing proteins potential therapeutic targets for inflammatory diseases?

Cell death is critical for maintaining homeostasis in multicellular organisms; too much can result in pathologies such as neurodegeneration, whereas too little can lead to the accumulation of malignant cancerous cells. Cell death can be either active, where the cell participates in its own destruction or passive, for example, when a cell undergoes irreparable physical damage.^1^ The most biochemically well-characterised form of cell death is apoptosis, an active process in which cysteine-dependent aspartate-directed proteases (caspases) are activated in response to extracellular stimuli or internal damage culminating in a form of cell death defined by distinct molecular events and characteristic changes in the morphology of the dying cell. Recently, a number of actively regulated non-apoptotic mechanisms of cell death have emerged, including necroptosis, pyroptosis and ferroptosis, which have been comprehensively reviewed elsewhere.^2, 3^ Here, we focus on those mechanisms of cell death arising following stimulation of death receptors, broadly termed the ‘extrinsic pathway'. For authoritative reviews on mitochondrial-mediated ‘intrinsic' cell death, we direct the Reader elsewhere.^4, 5^ Central to receptor-mediated cell death pathways are proteins containing ‘death–fold superfamily' interaction motifs such as the death domain (DD), caspase activation and recruitment domain (CARD), pyrin domain and the death effector domain (DED).

The DED-containing proteins, which are key decision makers in determining the life and death of cells, are the primary focus of this review. We will first introduce the main members of the DED protein family and discuss advances in the understanding of the assembly and stoichiometry of death receptor complexes. We will then summarise the recent literature surrounding the regulation of these complexes and consider the role of these proteins in disease.

## The DED Proteins

**Figure 1:**
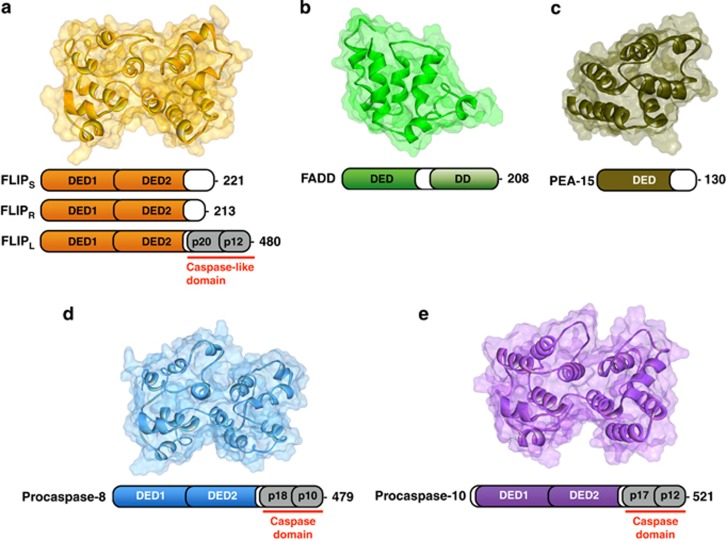
Schematic representations of the structures of DED proteins (ribbon format with transparent solvent-accessible surface area) along with their complete linear domain organisation. Protein structures: (**a**) Homology model of human FLIP DEDs^48^ (**b**) NMR solution structure of FADD DED (PDB ID: 2GF5) ^7^ (**c**) NMR solution structure of PEA-15 DED (PDB ID: 2LS7)^191^ (**d**) Homology model of procaspase-8 DEDs^48^ (**e**) Homology model of procaspase-10 DEDs generated using the I-TASSER web server^192^

The death-fold motif is characterised by its globular structure containing six amphipathic *α*-helices that run anti-parallel in *α*-helical bundles.^6^ When folded, a conserved hydrophobic core forms, although differences in helical length and residue distribution give rise to significant variations between the different sub-families.^6, 7, 8, 9^ The DED death-fold sub-family consists of procaspases-8 and -10, FLIP, FADD, DEDD, DEDD2, and PEA-15 (Figure 1). FADD, DEDD, DEDD2 and PEA-15 contain a single DED, whereas FLIP, procaspase-8 and procaspase-10 each have tandem DEDs. Procaspases-8 and -10 have catalytically active domains in the regions C-terminal to their tandem DEDs; whereas the long splice form of FLIP, FLIP_L_, has a pseudo-caspase domain C- terminal to its tandem DEDs, in which the cysteine residue critical for enzymatic activity is absent. Shorter splice forms of FLIP (FLIP short, FLIP_S_, and FLIP Raji, FLIP_R_) arising through alternative mRNA splicing lack the pseudo-caspase domain, but contain the tandem DEDs.^10^

Procaspase-8 is a highly conserved protease, displaying ~20% sequence similarity to its *Caenorhabditis elegans* homolog CED-3.^11^ Eight splice forms of procaspase-8 have been identified at the mRNA level, although only two of these, procaspases-8A and 8B are expressed as functional proteases.^12^ Additionally, a long splice form (procaspase-8L), which contains a 136-bp insert between exons 8 and 9 encoding an early stop codon, contains both DEDs but lacks a functional catalytic domain; it is found in undifferentiated cells and neoplasms and has been reported to act in a dominant negative manner to inhibit apoptosis.^13, 14, 15^

Procaspase-10 is also expressed as multiple splice forms: procaspases-10A, B, D and G. All contain tandem DEDs and proteolytic domains except G, which is truncated and only consists of the DEDs.^16, 17, 18^ *In vitro* studies have shown that procaspase-10 is activated by induced proximity in a manner similar to procaspase-8.^19^ Despite the similarity between procaspase-10 and procaspase-8, whether procaspase-10 can initiate death receptor-mediated apoptosis in the absence of procaspase-8 remains controversial,^20, 21, 22^ although they share common substrates, notably BID and RIPK1.^23, 24^ However, no ortholog of the gene encoding procaspase-10 (*Casp10*) is present in the mouse genome,^25^ suggesting that procaspase-10 is not required for activation of the extrinsic apoptotic pathway. Despite this, expression of both procaspase-8 and procaspase-10 is frequently downregulated in cancer.^26, 27, 28^ Interestingly, the genes encoding procaspase-8 (*Casp8*), procaspase-10 and FLIP (*CFLAR*) are present at the same loci (2q33-q34) and clearly evolved via gene duplication events. Evolutionary studies have identified the predecessors of *Casp8*, *Casp10* and *CFLAR* in fish.^29^

FADD is an adaptor protein containing a DD which allows it to associate with the DDs of TRAIL- R1, TRAIL-R2, CD95 and TRADD, and a DED, which enables it to recruit other DED-containing proteins, namely procaspase-8, procaspase-10 or FLIP. As with most other components of the extracellular apoptosis signalling pathways, FADD is highly evolutionarily conserved.^30^ As a protein linking death receptors to death initiators, it is not only a key player in cell death, but also has reported roles in non-apoptotic processes. For example, FADD has been identified in the nucleus and has been postulated to have functions in regulating cell cycle progression,^31, 32^ NF-*κ*B activity,^33^ autophagy,^34^ cytokine signalling^35, 36^ and T-cell activation.^37^ Indeed, roles beyond core apoptosis signalling have also been identified for FLIP, procaspase-8 and procaspase-10, and frequently these roles involve complex interactions between these proteins and FADD.

## Assembly of the Death-inducing Signalling Complex (DISC)

The death receptors TRAIL-R1 (DR4), TRAIL-R2 (DR5) and CD95 (Fas) are specialised members of the TNF receptor superfamily and are key mediators of apoptosis triggered by ligands expressed by cells of the immune system, namely TRAIL (TNF-related apoptosis-inducing ligand), which activates TRAIL-R1 and TRAIL-R2, and CD95L (FasL), which activates CD95. Following extracellular ligand binding, pre-associated TRAIL-R1, TRAIL-R2 and CD95 trimers, cluster through interactions between their intracellular DDs.^38, 39^ The DD of FADD can then interact with the cytoplasmic DDs of the death receptors, after which its DED becomes available for protein–protein interactions with other DED proteins, thereby creating a platform for assembly of the DISC.

### Death domain interactions

**Figure 2:**
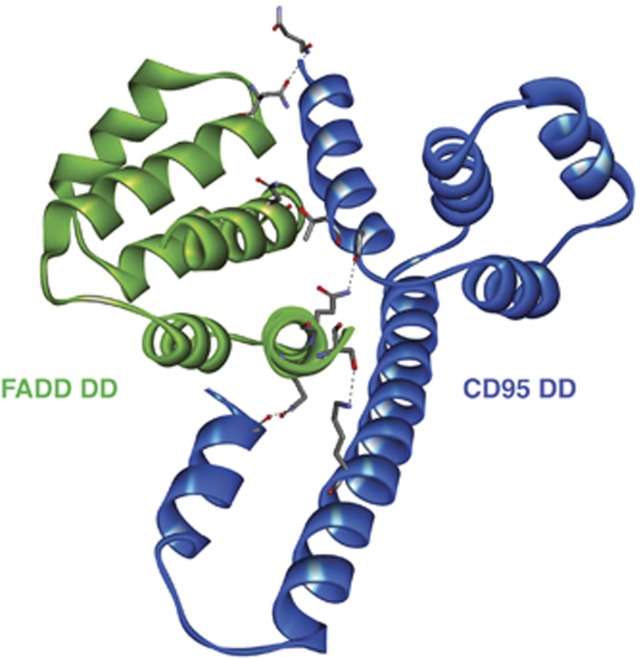
Crystal structure of the human CD95–FADD DD heterodimer. CD95 DD (blue ribbon) binds to FADD DD (green ribbon) via several key interacting residues (highlighted as grey sticks) (PDB ID: 3EZQ).^40^ Hydrogen bonds are depicted as black dotted lines. These heterodimers oligomerise into higher order structures

The interactions between the CD95 and FADD DDs have been described (Figure 2). Scott *et al.*^40^ reported a 2.7 Å resolution co-crystal structure, which suggests that CD95 and FADD bind in dimeric units, that is 2 × FADD-DD interacting with 2 × CD95-DD. These units are then proposed to further associate into tetrameric structures (4 × FADD-DD to 4 × CD95-DD), although *in vivo*, the dimeric form is favoured. They report that, following CD95 receptor activation, CD95 undergoes a conformational change, exposing its hydrophobic core and revealing a multitude of interaction surfaces capable of binding the DD of FADD. This represents a possible safety mechanism whereby the apoptotic cascade only proceeds when sufficient CD95 DDs cluster. However, the FADD interaction sites in the CD95 DD predicted by this model do not correlate with mutations observed in patients with autoimmune lymphoproliferative syndrome, a disease defined by mutations in CD95 which prevent DISC formation.^41^ In a different study, Wang *et al.*^42^ used electron microscopy to visualise CD95 DD–FADD DD interactions and observed that they bore a striking resemblance to the PIDD DD–RAIDD DD complex, being principally composed of 5 × CD95 DDs and 5 × FADD DDs layered together. This stoichiometry is in agreement with data by Esposito *et al.*,^43^ who also reported a ratio of 5 × CD95:5 × FADD together with some 6 × CD95:5 × FADD and 7 × CD95:5 × FADD ratios, but *not* the 4 × CD95:4 × FADD suggested by Scott *et al.* Disparities between these models could be explained by the different conditions used for protein crystallisation; however, the models proposed by Wang *et al.* and Esposito *et al.* are supported by the fact that they account for mutations frequently seen in autoimmune lymphoproliferative syndrome. Most disease-causing mutations present in autoimmune lymphoproliferative syndrome patients reside in the DD of CD95,^41^ resulting in an inability to bind FADD and form a DISC. Mapping these mutated residues onto the structure proposed by Wang *et al.* reveals that they reside on the exposed surface of the DD and would be likely to prevent the binding of FADD.^42^

### DED interactions

**Figure 3:**
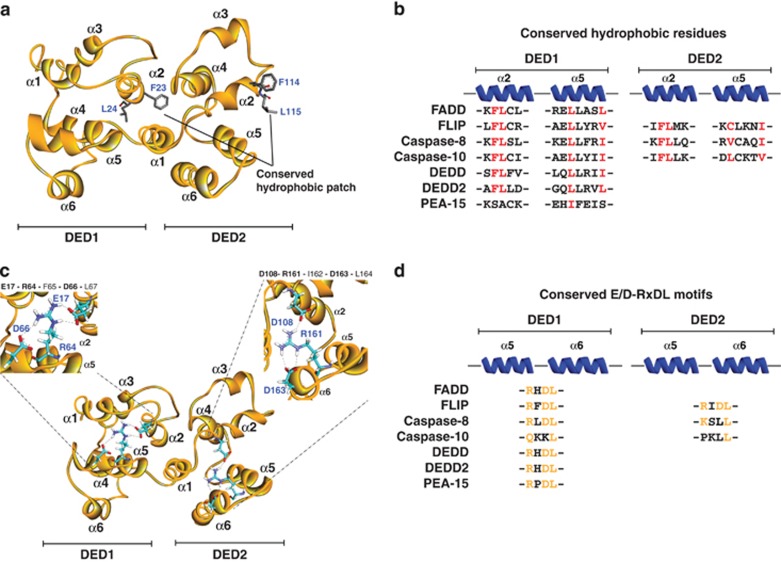
Conserved features of the DED protein family. (**a**) Homology model of the tandem DEDs of human FLIP (orange ribbon) depicting the main conserved residues part of the hydrophobic patch (highlighted in grey), which are essential for inter- and intra-DED–DED interactions. (**b**) Sequence alignment of residues that form part of the hydrophobic patches, which are highly conserved amongst DED proteins. (**c**) Homology model of human FLIP DEDs (orange ribbon) with a close-up view of the two E/D-RxDL motifs. Only residues that form the charged triad motifs (highlighted in cyan) are shown. Hydrogen bonds are highlighted in black dotted lines. (**d**) Sequence alignment of the E/D-RxDL motifs which are conserved amongst DED proteins

**Figure 4:**
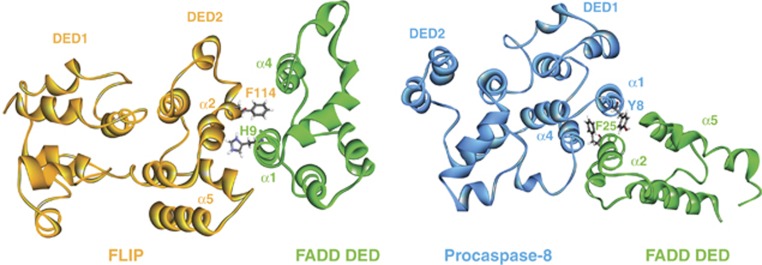
Binding modes of FLIP–FADD and procaspase 8–FADD interactions. FLIP binds to the DED of FADD using its DED2, whereas procaspase-8 binds to FADD using its DED1. The main residues that are important for the interactions are shown in grey: FLIP uses its F114 residue on the *α*2 helix to bind into a groove between *α*1/*α*4 helices in the DED of FADD with a reciprocal interaction from FADD H9 into the *α*2/*α*5 hydrophobic patch of FLIP; procaspase-8 uses the Y8 residue on its *α*1 helix to bind into the hydrophobic patch between the *α*2/*α*5 helices in FADD, with a reciprocal interaction from FADD F25 into the *α*1/*α*4 groove in procaspase-8

In addition to death receptor:FADD DD interactions, FADD has been reported to self-associate through its DED, which stabilises its association with the death receptor. Sandu *et al.*^44^ identified a ‘hydrophobic patch' (F25, L28 and K33) as the critical surface for FADD-FADD interactions and not an RxDL motif as had previously been reported. Such interactions between FADD molecules generates higher order complexes of FADD and death receptors that may be the basis of the SPOTS (signaling protein oligomerisation transduction structures), which have been reported to form soon after CD95 receptor activation.^45^ The RxDL motif is found in both DEDs of viral FLIP MC159 and is critical for its ability to be recruited to the DISC and inhibit apoptosis.^9, 46, 47^ However, this appears not to be the case for murine FLIP, for which the hydrophobic patch was instead found to be indispensable for DISC recruitment and apoptosis inhibition.^47^ MC159 was also reported to interact with the RxDL motif of FADD, blocking FADD self-association and preventing the formation of a competent caspase-recruiting platform.^8^ The role of the RxDL motif may differ for cellular and viral forms of FLIP. Our recent data suggest that this motif is important for human FLIP's anti-apoptotic function; however, not because of its direct involvement in mediating inter- and intra-molecular interactions, but rather because it controls the spatial orientation of the hydrophobic patch defined by the α2 and α5 helices of FLIP's DEDs (Figure 3); our data and those of others indicate that it is this hydrophobic patch that mediates intra-molecular interactions between FLIP's tandem DEDs and inter-molecular interactions between FLIP and FADD and procaspase-8 (Figure 4).^48^

### Emerging models of DISC assembly at the level of DED interactions

Two independent studies proposed a novel model of DISC assembly after finding that FADD is sub-stoichiometric at the DISC compared with death receptors and caspase-8, with three to five receptors and as many as nine caspase-8 molecules for every FADD molecule recruited to the complex.^49, 50^ DED-containing proteins interact with themselves and one another in a homotypic manner through their DEDs, so both groups proposed that one FADD molecule could recruit multiple DED-only proteins (procaspase-8, procaspase-10 or FLIP) as ‘chains'. In support of this model, formation of caspase-8 chains was observed in single cell studies using fluorescently tagged caspase-8. Such DED ‘filaments' have been described before for caspase-8 and FADD;^46, 51^ however, their physiological relevance is questionable as they are generated in cells expressing supra-physiological levels of each DED protein.

By creating models of FLIP and procaspase-8 DEDs based on the published structure of vFLIP MC159,^8^ we used the NMR structure of FADD^7^ to perform docking experiments between the DEDs of the three proteins. These modelling experiments suggested that each protein pair could potentially interact in two distinct orientations, which involved mainly hydrophobic interactions between the α2/α5 surface (the aforementioned hydrophobic patch) in one DED and α1/α4 surface of the adjacent DED.^48^ Subsequent mutagenesis studies revealed that FLIP and procaspase-8 have differential affinities for the two available interaction surfaces of the FADD DED. FLIP preferentially binds to the *α*1/*α*4 surface of FADD's DED, whereas procaspase-8 binds to FADD's *α*2/*α*5 surface (Figure 4). Our analysis of the stoichiometry of the TRAIL-R2 DISC was not in agreement with the caspase chain models described above: in our study, sub-apoptotic DISC stimulation resulted in an approximate 1:1:1 ratio of FADD:caspase-8:FLIP; while at higher levels of DISC stimulation, there was more caspase-8 than FADD or FLIP, although there remained approximately one FADD molecule for every two molecules of FLIP/caspase-8.

### Activation of caspase-8

**Figure 5:**
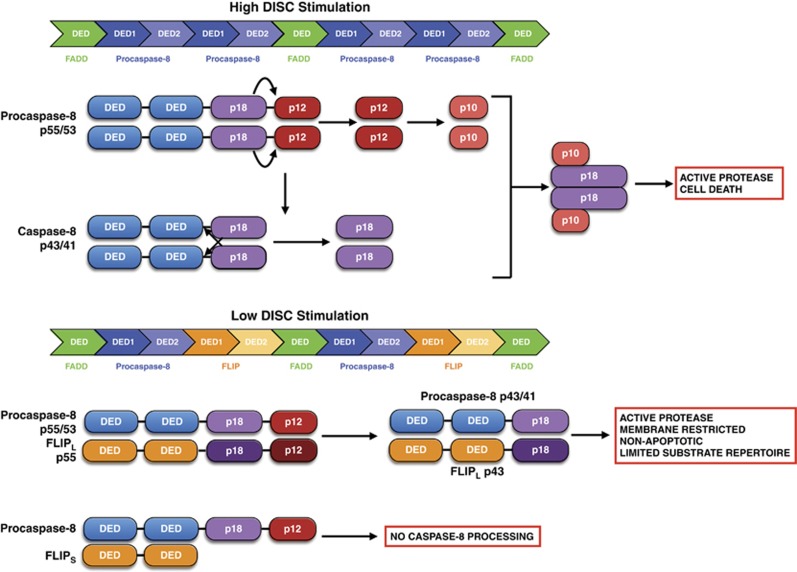
Processing of procaspase-8. When two procaspase-8 molecules are co-recruited to the DISC via their DEDs, their caspase domains undergo conformational changes that exposes the enzymatic activity necessary for cleavage of the C-terminal portion of the caspase, liberating a p12 subunit, which is subsequently processed to the small p10 catalytic subunit. This initial processing step may occur in an inter-dimer manner between adjacent procaspase-8 dimers rather than the intra-dimer manner depicted.^63^ The 41/43 kDa caspase-8 intermediates cleave one another in a *trans*-catalytic manner in the region between their DEDs and the large p18 catalytic subunit. The two molecules of pro-caspase-8 that are subsequently released associate with the two p10 subunits to form the active protease.^57^ At lower levels of DISC stimulation or when FLIP is highly expressed, FLIP/caspase-8 heterodimers assemble at the DISC via interactions between their DEDs and those of FADD.^48^ The pseudo-caspase domain of FLIP_L_ is able to induce the conformational change in procaspase-8's caspase domain that is necessary to create its active site.^59^ The FLIP_L_:caspase-8 heterodimer is processed between the p18 and p12 subunits of both proteins, but is unable to be further processed owing to FLIP_L_'s lack of enzymatic activity, and this heterodimer is unable to activate apoptosis. In the case of FLIP_S_, heterodimerisation fails to activate procaspase-8 as the initial conformational change cannot take place in procaspase-8's caspase domain

The current model of procaspase-8 activation is that 53/55 kDa procaspase-8 zymogens are recruited to FADD as monomers via their DEDs leading to dimerisation of the procaspases, initially via their DEDs (Figure 5).^52, 53, 54, 55, 56^ Dimerisation of the caspase domains then occurs and results in conformational changes that reveal the enzymatic activity necessary for intra-molecular cleavage of the C-terminal portion of the caspase, liberating a p12 subunit (subsequently processed to the small p10 catalytic subunit) and simultaneously stabilising the dimer. Next, the 41/43 kDa caspase-8 intermediates in the dimer cleave one another in a *trans*-catalytic manner in the region between their DEDs and the large p18 catalytic subunit. The two molecules of p18-caspase-8 that are subsequently released associate with the two p10 subunits to form the active protease.^57^ These two steps are critical, as cleavage in the absence of dimerisation does not result in an active protease.^58^

Procaspase-8 can also heterodimerise with FLIP at the DISC. In the case of FLIP_S/R_, heterodimerisation fails to activate procaspase-8 as the initial conformational change cannot take place in procaspase-8's caspase domain;^59^ thus, FLIP_S/R_ effectively acts in a dominant negative manner (Figure 5). For FLIP_L_, heterodimerisation results in an active enzyme, as the pseudo-catalytic domain of FLIP_L_ is able to induce the conformational change in procaspase-8's caspase domain that is necessary to create the active site (Figure 5).^59^ Indeed, it appears that FLIP_L_'s pseudo-caspase domain is more efficient at inducing the conformational change in the dimer than the caspase domain of another molecule of procaspase-8.^60^ The FLIP:caspase-8 heterodimer remains tethered to the DISC because the second step of activation, cleavage between the DEDs and p18-subunits cannot occur because of FLIP_L_'s lack of enzymatic activity, and the heterodimer is unable to activate the apoptotic cascade. However, the FLIP_L_:caspase-8 heterodimer's enzymatic activity can cleave local substrates, most notably RIPK1, an important regulator of necroptosis (*see below*).^61^

Procaspase-8 dimerisation is required for formation of the active site.^55, 56^ However, as well as *intra*-dimer cleavage, procaspase-8 can be cleaved in an *inter*-dimeric manner (i.e., dimers acting in a *trans* manner).^54, 62^ To define the relative contributions of each of these modes of caspase-8 activation, Kallenberger *et al.*^63^ used single cell analysis and mathematical modelling. They suggest a model in which the cleavage of procaspase-8 between the enzymatic p18 and p10 domains occurs in an *inter*-dimeric manner, while cleavage between the pro-domain and p18 domains occurs in an *intra*-dimer manner.^63^ This model implies that only formation of adjacent procaspase-8 dimers will result in full procaspase-8 processing in each dimer; thus, a FLIP:caspase-8 heterodimer may also inhibit full activation of an adjacent caspase-8:caspase-8 homodimer.

### TNFR1 complexes I and II

**Figure 6:**
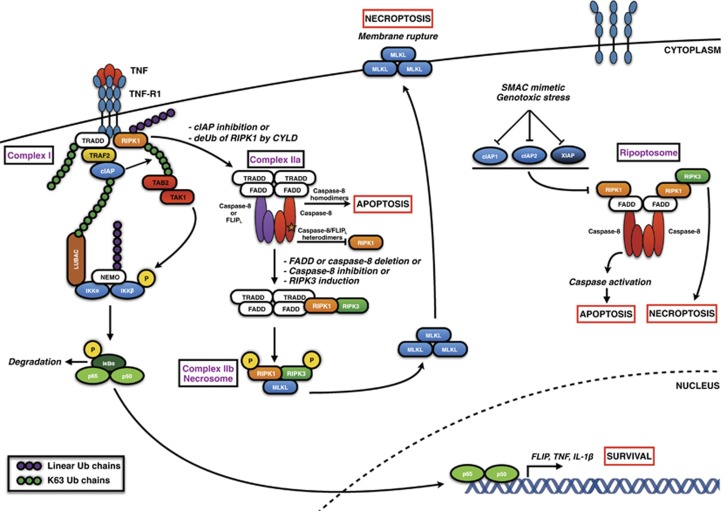
DED proteins in TNF signalling. Binding of TNF to TNFR1 stimulates the recruitment of the adaptor molecules TRADD and RIPK1. TRAF2 binds to TRADD, which in turn associates with cIAP1/2 forming Complex I.^64^ The E3 ligases cIAP1/2 then conjugate ubiquitin chains to various elements of Complex I enabling the recruitment and stabilisation of LUBAC.^69, 70^ NEMO and IKK associate with LUBAC, and IKK is phosphorylated by TAK1, resulting in its proteasomal degradation and allowing subsequent translocation of the NF-κB subunits p65/p50 to the nucleus. However, in conditions of cIAP inhibition (e.g., SMAC mimetics) or deubiquitination of RIPK1 by the DUB CYLD, Complex I can dissociate from the membrane and recruit FADD, FLIP and procaspase-8 to form Complex IIa.^193, 194^ Depending on the composition of Complex IIa, RIPK1 is cleaved and apoptosis ensues. If FADD or procaspase-8 are deleted, caspase-8 activity is inhibited (e.g., by caspase inhibitor-encoding viruses) or RIPK3 is induced (e.g., following RIPK1 autophosphorylation), Complex IIb (or the necrosome) is formed which facilitates the phosphorylation of the pseudokinase MLKL by RIPK3.^83, 195^ MLKL then oligomerises and translocates to the plasma membrane, binds to phosphatidylinositol phosphates, disturbing membrane integrity and leading to necrotic cell death.^83, 84, 196, 197, 198, 199^ Under normal physiological conditions, RIPK1 is ubiquitinated by cIAP1/2 and degraded;^70^ however, under conditions where IAPs are depleted, such as SMAC mimetic treatment or genotoxic stress, RIPK1 is available to bind FADD, procaspase-8, FLIP and RIPK3. This complex termed the ‘Ripoptosome' can result in either apoptosis or necroptosis depending on the levels of FLIP_L_, FLIP_S_ and procaspase-8 recruited.^86, 92^

Seminal work by Micheau and Tschopp^64^ showed that following TNF*α* engagement, TNF receptor 1 (TNFR1) trimerises and recruits the adaptor protein TRADD in a DD-dependent manner. TRAF2, a RING domain-containing E3 ligase, is recruited to TRADD and forms a platform for the recruitment of cIAP1 and cIAP2.^65, 66^ RIPK1 is also recruited to form TNFR1 Complex I, and the cIAP proteins then conjugate K11- and K63-linked polyubiquitin chains to RIPK1 enabling its interaction with the IKK complex and activation of the NF*κ*B signalling pathway; this in turn results in transcription of genes, which predominantly encode pro-survival (including FLIP and cIAP1) and pro-inflammatory proteins (Figure 6).^67, 68, 69, 70, 71, 72, 73^ In 2009, Tokunaga *et al.*^74^ found that mice deficient in components of the linear ubiquitin chain assembly complex (LUBAC), specifically HOIL-1, are defective in TNF*α*-induced NF*κ*B activation.^75^ Utilising a modified tandem affinity purification technique, Haas *et al.*^75^ showed that the LUBAC is recruited to the TNFR1 signalling complex through cIAP-generated ubiquitin chains. Subsequent studies identified NEMO and RIPK1 as substrates of linear ubiquitination by LUBAC.^76^ The LUBAC appears to stabilise the TNFR1 signalling complex, prolonging recruitment and retention of cIAP1, cIAP2, TRAF2, RIPK1 and TAK1.^75^ The central importance of cIAP1/2 in preventing TNF*α*-induced NF-*κ*B activation is supported by evidence from cIAP1/2-null genetic models which die following exposure to TNF*α* to a much greater extent than loss of RIPK1 or LUBAC alone.^77, 78, 79, 80^ Deubiquitination of RIPK1 by CYLD^81^ stimulates the dissociation of Complex I into a secondary cytoplasmic Complex IIa where RIPK1 and/or TRADD recruit FADD via their DDs. FADD in turn recruits procaspase-8 and FLIP into this complex in a manner analogous to that described above for the DISC.^64, 82^ As FLIP is an NF-*κ*B target gene, prior activation of Complex I upregulates its expression, resulting in its recruitment to Complex II and regulation of procaspase-8 processing in the manner described above. Importantly, the formation of a caspase-8/FLIP_L_ heterodimeric enzyme at Complex IIa cleaves RIPK1, which otherwise auto-phosphorylates and interacts with RIPK3 to form the necrosome. The necrosome in turn initiates programmed necrosis (termed necroptosis) by triggering oligomerisation of MLKL (mixed lineage kinase domain-like), which then localises to the plasma membrane and disrupts its integrity.^83, 84^ Thus, formation of the caspase-8/FLIP_L_ heterodimer in Complex IIa blocks both apoptosis by preventing procaspase-8 homodimerisation and necroptosis by blocking RIPK1/RIPK3-mediated necroptosis.^85^ As is the case for other DED protein-containing complexes, caspase-8/FLIP_S_ heterodimers in Complex IIa lack catalytic activity, and although their formation inhibits caspase-8-mediated apoptosis, they have been reported to actually promote RIPK-mediated necroptosis by inhibiting caspase-8-mediated cleavage of RIPK1.^86^ A general view is that compared with apoptosis, necroptosis is highly pro-inflammatory owing to the release of pro-inflammatory cytokines^87^ and damage-associated molecular patterns.^88, 89^ However, recent findings show that necroptosis can actually reduce certain pro-inflammatory responses, while CD95-mediated apoptosis has been shown to stimulate release of immuno-stimulatory cytokines.^90, 91^

### The Ripoptosome

In the last few years, a new cytosolic DED-containing complex has been described, termed the ‘Ripoptosome' (Figure 6). During normal cellular homeostasis, RIPK1 exists in a closed configuration and cannot bind FADD via its DDs.^86, 92^ In its open configuration, RIPK1 is usually ubiquitinated by cIAP1/2 and degraded in a proteasome-dependent manner. However, cIAPs can themselves be degraded in response to genotoxic stress (e.g., in response to DNA-damaging chemotherapeutics) or more specifically in response to IAP antagonists (also known as SMAC mimetics as they mimic the activity of the endogenous IAP inhibitor, SMAC). In the absence of cIAPs, RIPK1 is phosphorylated and transitions into an open configuration, allowing it to more readily bind FADD, procaspase-8, FLIP and potentially RIPK3.^93^ This complex has been termed the Ripoptosome, and it can initiate apoptosis or necroptosis in a manner similar to that described above for TNFR1 Complex IIa depending on its composition.^86, 92^

### DED proteins in embryonic development

This pro-survival role of caspase-8 in suppressing necroptosis explains the results of several genetic experiments that stemmed from older observations that caspase-8-null, FADD-null and FLIP-null mice die at E10.5 with similar phenotypes.^94, 95, 96^ This has now been attributed to the loss of FADD or caspase-8 leading to unrestrained RIPK1/3-mediated necroptosis during mid-gestation, whereas loss of FLIP results in unrestrained caspase-8-mediated apoptosis at this time. Thus, combined deletion of FADD or caspase-8 with RIPK3 can prevent necroptosis and rescue the embryonic lethal phenotype of the FADD-null and caspase-8-null genotypes.^85, 97^ However, to rescue the embryonic lethality of FLIP-null animals, loss of FADD *and* RIPK1 or RIPK3 is required, as in this case, both FADD-mediated apoptosis and RIPK3-mediated necroptosis must be blocked. In related observations, tissue-specific deletion of caspase-8 in skin^98^ or the gut^99, 100^ (but not in liver,^101^ myeloid cells^101^ or the heart^102^) resulted in severe inflammation owing to unrestrained inflammatory signalling. *RIPK3* ablation in *Casp8*^*−/−*^ gut safeguards these tissues from the inflammation observed with *Casp8* deletion alone, but *FLIP* deletion cannot be rescued by simultaneous *RIPK3* deletion, implying that the cell death in *FLIP*^*−/−*^ *gut* is most likely apoptotic, not necroptotic.^100^ Interestingly, in both *Casp8*-deleted and *FLIP*-deleted skin, elevated levels of TNF*α* were produced, and co-administration of a TNF*α*-neutralising antibody prevented inflammation and death, implying that this phenotype is TNF*α*-driven.^100^

### Role for DED protein complexes in autophagy

Evidence is now emerging that DED proteins can also regulate autophagy. Young and colleagues^103^ provide compelling evidence that FADD co-localises with LC3-positive autophagosomes capable of recruiting procaspase-8 and forming an intracellular DISC.^104^ Depending on the cellular context, autophagy can promote survival or death (reviewed by White^105^). There is strong evidence that autophagy can promote caspase-dependent and -independent cell death;^106^ the intracellular DISC provides one potential complex through which this can occur. Proteasome inhibition has been shown to activate caspase-8 in a manner that requires the induction of autophagy and the presence of Atg5 and FADD.^107^ Another study reported that a FADD/caspase-8/Atg5:12/RIPK1 complex forms on autophagosomal membranes in T cells and cleaves RIPK1, limiting autophagy and necroptosis and ultimately initiating apoptosis.^108, 109, 110^

Cellular and viral forms of FLIP can also attenuate autophagy through inhibitory binding to Atg3, a key component of the LC3 conjugation system.^111^ Inhibition of Atg3 by FLIP can be relieved by peptides from either its DED1 *α*2-helix or the DED2 *α*4-helix, suggestive of a more promiscuous, non-discriminatory mode of binding than that between FLIP and FADD.^48^ Through an RNAi library screening approach, Lamy *et al.*^112^ identified caspase-10 as critical for survival of multiple myeloma cells; the authors found that caspase-10 forms a proteolytically active complex with FLIP_L_, which constitutively cleaves and inactivates BCLAF1. As BCLAF1 displaces Beclin-1 from BCL-2 to promote autophagic cell death,^113^ this caspase-10/FLIP_L_ complex blocks this mode of cell death. These results suggest that pharmacological inhibition of caspase-10 may afford therapeutic benefit to multiple myeloma patients by inducing autophagic cell death. Interestingly, despite their similarities, this effect was not observed when caspase-8 was depleted.^112^ The upstream mechanism that triggers formation of this caspase-10/FLIP_L_ heterodimer and the identities of other members and substrates of this complex are currently unknown.

## Posttranslational Regulation of DED Proteins

### Ubiquitination events at the DISC

Given the swiftness of a cell's response to apoptotic stimuli, it is unsurprising that cells have numerous mechanisms to tightly regulate the expression and function of the key decision makers. In a series of studies, work from the Ashkenazi laboratory has revealed the critical role of ubiquitination in the activation of caspase-8 at the DISC. Firstly, they showed that caspase-8 is polyubiquitinated with K63-linked chains by cullin 3 (CUL3) at the DISC in response to either TRAIL-R1 or TRAIL-R2 stimulation.^114^ Moreover, silencing CUL3 inhibited caspase-8 processing at the DISC, suggesting a role for CUL3-mediated ubiquitination in regulating caspase-8 activation. In mapping studies, the C-terminus of caspase-8 was identified as the region of ubiquitination. Furthermore, aggregation and activation of polyubiquitinated caspase-8 is facilitated by p62,^115^ a protein known to bind to ubiquitin, that moves caspase-8 into ubiquitin-rich foci.^114^ However, it is unknown whether translocation into these foci is necessary for caspase-8′s activation, and the implications this has for the observation that caspase-8 and p62 are both found in intracellular DISCs and autophagosomal membranes is unclear.^103, 104^ In subsequent work, the same group found that cytosolic p43 and p18 fragments of caspase-8 are degraded in a proteasome-dependent manner. In this latter study, caspase-8 was reported to be conjugated by degradative K48-linked ubiquitin chains on its p18 fragment by another E3 ligase, TRAF2.^116^ Rather than increasing its activation as is the case for CUL3-mediated K63-ubiquitination, TRAF2 acts to degrade the pool of activated caspase-8, decreasing the propensity of the cell to commit to apoptosis. Caspase-8-processed FLIP_L_ has also been reported to interact with TRAF2, promoting activation of the NF-*κ*B transcriptional pathway, but it is not yet clear whether these observations are related.^117^

### Regulation of FLIP by the UPS

An additional layer of control of death receptor-mediated apoptosis is achieved by the ubiquitination of FLIP. Similar to the anti-apoptotic BCL-2 family member MCL-1, FLIP_S_ is an extremely short-lived protein that is rapidly turned over through the ubiquitin-proteasome system.^118^ FLIP_S_ is ubiquitinated on K192 and K195 in DED2 and, between these two lysine residues at position 193 is a serine residue which, when phosphorylated, inhibits the ubiquitination of the adjacent lysines.^118, 119^ Notably, mutational studies showed that ubiquitin-deficient mutants of FLIP_S_ had increased half-lives (as expected) but were still recruited to the DISC, retaining their anti-apoptotic ability. In further work, the same lab identified PKC*α*/*β* as the key mediators of FLIP_S_ phosphorylation on S193.^119^ FLIP_L_ is less labile than FLIP_S_, although it too is turned over relatively rapidly, with a typical half-life of 2–3 h.^118^ A similar interplay between phosphorylation and ubiquitination is true for FLIP_L_, where ROS production induces the phosphorylation and subsequent ubiquitination and degradation of FLIP_L_.^120^ Moreover, K195 is a site of ubiquitin conjugation on FLIP_L_ in response to hyperthermia.^121^ Our group has identified a role for the DNA repair protein Ku70 in regulation of FLIP ubiquitination. Ku70 forms a complex with FLIP protecting it from ubiquitination and subsequent degradation. This complex is regulated by the acetylation of Ku70 and thus can be manipulated pharmacologically by histone deacetylase inhibitors leading to rapid degradation of FLIP.^122^ Chang *et al.*^123^ reported that following TNF*α* stimulation, JNK is activated which in turn activates the E3 Ubiquitin ligase Itch, resulting in FLIP_L_ ubiquitination and subsequent proteasomal degradation. This apparent link between FLIP and Itch was partly confirmed by other studies, including Panner *et al.*,^124, 125^ who identified a PTEN-Akt-Itch pathway controlling FLIP_S_ stability and degradation.

## DED Complexes in Mammalian Host Defence

### Antiviral immunity

DED-containing protein complexes are critical for innate immune reactions and can assemble to induce apoptosis in response to viral infection, generally in a mitochondrial antiviral signalling adaptor (MAVS)-dependent manner. Typically, cytosolic viral RNA is recognised by one of the three RIG-1-like receptors (RLRs) retinoic acid-inducible gene I (RIG-1), melanoma differentiation-associated gene 5 (MDA5) or laboratory of genetics and physiology 2 (LGP2). RIG-1 and MDA5 undergo conformational changes upon binding to viral RNA, exposing an N-terminal CARD that interacts with and activates MAVS,^126, 127^ which in turn activates a type I interferon response.^126^

**Figure 7:**
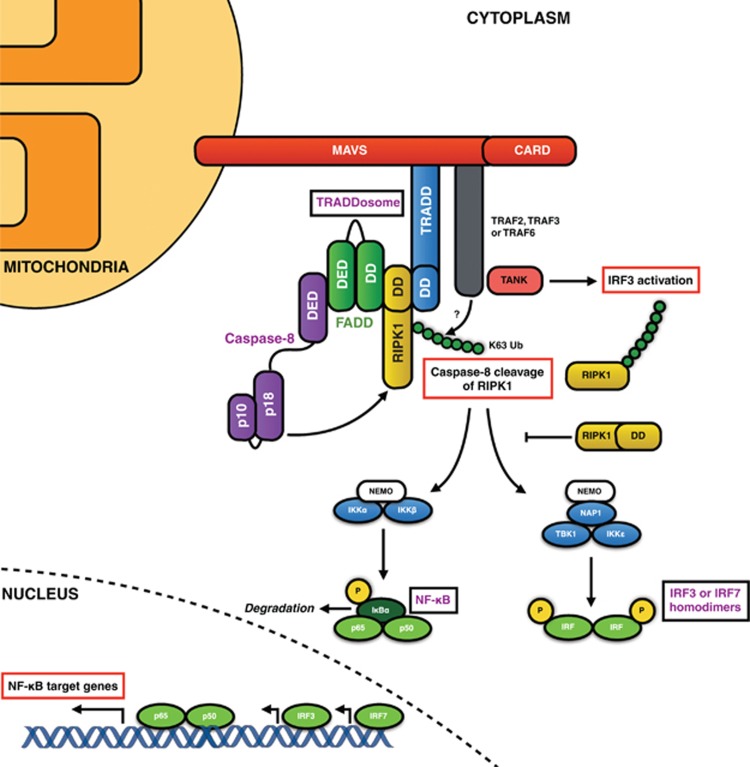
DED proteins in MAVS signaling. Following viral infection of cells, viral RNA is detected by CARD-containing RIG-1-like receptors, for example, RIG-1 and MDA5. RIG-1 binds the dsRNA, exposing its normally hidden CARD domains. K63 ubiquitin chains are conjugated to the CARDs, facilitating the assembly of a complex composed of four polyubiquitin chains and four RIG-1 molecules (not shown).^200, 201^ This in turn induces the formation of prion-like aggregates of MAVS, which strongly activate IRF3.^202, 203^ These MAVS aggregates form a platform which can recruit TRAF2, TRAF3 and TRAF6.^203^ TRADD also binds MAVS followed by TANK and TBK2, activating antiviral IRF3.^129, 203^ However, TRADD can also recruit RIPK1, FADD and caspase-8, a complex dubbed the ‘TRADDosome'. Caspase-8 cleaves RIPK1 and the resulting RIPK1 fragment can inhibit IRF3, ceasing the antiviral response.^204^ RIPK1 is conjugated by K63 ubiquitin chains inducing two distinct signaling pathways from the TRADDosome: firstly, NF-*κ*B signaling through NEMO, IKK*α* and IKK*β*, and secondly NEMO can interact with NAP1, TBK1 and IKKɛ to activate IRF3 or IRF7.^205, 206, 207, 208^ In addition to caspase-8, the TRADDosome can also recruit FLIP (not shown) and may under certain conditions, trigger cell death

FADD and RIPK1 can interact with MAVS (Figure 7),^128^ and FADD- and RIPK1-deficient cells are hypersensitive to viral infection owing to an inability to induce the transcription of key antiviral genes.^35^ Tschopp and co-workers elucidated the mechanistic basis of these findings and found that the antiviral RIG-1 signalling pathway bears a striking resemblance to the TNFR1 pathway.^64, 129^ They propose that following viral infection, a complex termed the ‘TRADDosome' (composed of TRADD, RIPK1 and FADD) forms on the mitochondrial membrane via MAVS. In this MAVS-located TRADDosome, RIPK1 can be K63 ubiquitinated by TRAF2/3 and recruit NEMO resulting in activation of IKK*α* and IKK*β* and subsequent phosphorylation and degradation of I*κ*B leading to NF-*κ*B activation. Finally, via FADD, the TRADDosome can recruit procaspases-8 and -10 and FLIP, potentially inducing cell death.^129^

### DED proteins and the inflammasome

**Figure 8:**
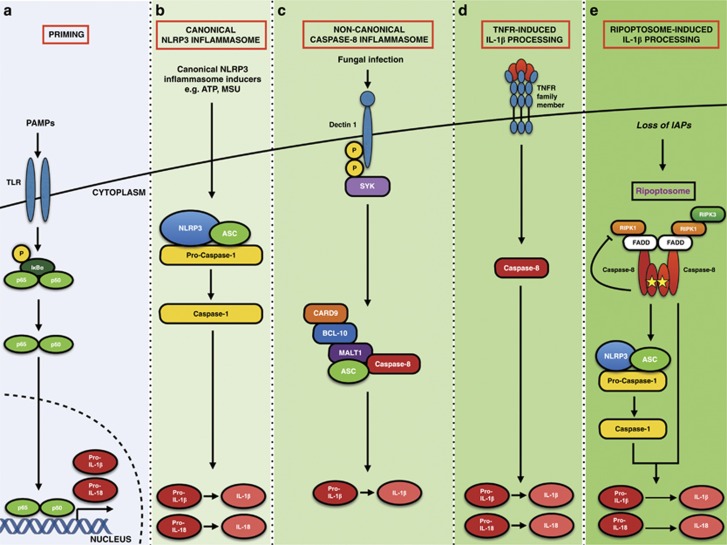
Involvement of DED proteins in the inflammasome. (**a**) Full activation of the inflammasomes requires a two-step process. Firstly, a priming signal is induced by a PAMP (such as LPS) activating a transcriptional cascade resulting in the *de novo* synthesis of inflammasome components such as NLRP3 and pro-IL-1*β*.^139^ Of note, subsequent work has shown that this is not always the case, as TLR-mediated priming of the NLRP3 inflammasome does not always require transcriptional upregulation of NLRP3.^209, 210^ (**b**) Assembly of the inflammasome components NLRP3, ASC and pro-caspase-1 occurs following a second signal. This can be any one of numerous different stimuli, for example, ATP activation of the P2X7 receptor, bacterial toxins, nigericin and silica.^211, 212, 213^ A subsequent efflux of potassium from the cell permits the components to form a functional canonical NLRP3 inflammasome where pro-caspase-1 is cleaved into its active form.^214^ Catalytically active caspase-1 cleaves pro-IL-1*β* and pro-IL-18 into their mature forms, which are then released from the cell.^211^ (**c**) Fungi and mycobacterium activate the *β*-glucan receptor dectin-1 resulting in the phosphorylation of cytoplasmic domain allowing the recruitment of SYK kinase.^215^ This elicits the formation of the non-canonical caspase-8 inflammasome, consisting of CARD9, BCL-10, MALT1, ASC and caspase-8. In this complex, active caspase-8 cleaves pro-IL-1*β* into its mature form which is released from the cell.^216^ (**d**) IL-1*β* and IL-18 can be processed directly by caspase-8 in an ASC-independent manner following ligation of members of the TNF receptor family, such as CD95, although the mechanism for this remains unclear.^146^ (**e**) The Ripoptosome forms upon loss of IAPs and can lead to activation of caspase-8, which can potentially cleave IL-1*β* directly or indirectly via caspase-1.^150, 217^

The inflammasome is a multi-protein oligomeric structure formed in macrophages and monocytes in response to inflammatory stimuli (Figure 8).^130^ Inflammasomes are comprised of a stimulus-specific sensor protein belonging to either the NLR, AIM2 or ALR family, the adaptor protein ASC and the inactive zymogen procaspase-1.^131^ Formation of the inflammasome leads to activation of caspase-1, which processes pro-IL-1*β* (and IL-18) to its mature form.^132, 133^ Different inflammasomes assemble in response to distinct stimuli, for example, the NLRP3 inflammasome forms in response to a plethora of pathogens, including influenza A,^134, 135^ *Klebsiella pneumoniae* and *Staphylococcus aureus*, in addition to endogenous danger signals such as ATP, uric acid crystals, nigericin and hyaluronan.^131^ The NLRC4 inflammasome reacts to bacterial flagellin and PrgJ, and the AIM2 inflammasome detects foreign dsDNA.^136, 137^ RIG-1 senses RNA viruses and forms a signaling complex with ASC, activating an inflammasome.^138^ Toll-like receptors (TLRs) detect pathogens by recognising pathogen membrane proteins (TLR4) or cytoplasmic nucleotides (TLR3). Full activation of inflammasomes requires two distinct stages: a ‘priming' signal 1, for example, from either a TLR agonist or a pro-inflammatory cytokine, that activates NF-*κ*B and upregulates pro-IL-1*β* expression,^139, 140^ followed by stimulus-specific inflammasome activation and processing of pro-IL-1*β*.^141, 142^

DED-containing proteins are emerging as players in inflammasome signalling. Caspase-8-dependent TLR4 signalling is critical for inflammasome assembly and IL-1*β* processing in glaucoma,^143^ and engagement of TLR3 or TLR4 can result in processing of IL-1*β* by caspase-8, independent of NLRP3 and caspase-1.^144^ Studies by Kanneganti and colleagues^145^ revealed that FADD and caspase-8 are obligatory for the correct priming and activation of both the canonical and non-canonical NLRP3 inflammasome, and that CD95 can induce IL-1*β* and IL-18 maturation in a caspase-8-dependent, but RIPK3-independent manner.^146^ However, work from other groups contest this: Allam *et al.*^147^ and Weng *et al.*^148^ show that caspase-8 is required only for TLR-induced inflammasome priming. A number of other studies show normal canonical NLRP3 inflammasome activation in caspase-8-deficient cells, refuting an essential role for caspase-8 in NLRP3 inflammasome activation.^149, 150, 151^ Together, these data suggest that caspase-8 may promote caspase-1 activity under certain conditions, but is not absolutely required for NLRP3 inflammasome activation.

FLIP_L_ has been shown to be involved in activation of the NLRP3 and AIM2 inflammasome and directly interacts with NLRP3, AIM2 and procaspase-1.^152^ In contrast, FLIP decreased IL-1*β* generation in response to SMAC mimetics and CD95 receptor activation, indicating that its role in regulating the inflammasome is context-dependent.^152^

### DED proteins and the adaptive immune system

It has long been known that caspase-8- or FADD-deficient T cells do not proliferate in response to T-cell receptor activation;^37, 153^ it had previously been thought that this was due to a defective ability to activate NF-*κ*B, but recent work has shown that it is due to induction of necroptosis.^154, 155^ Rescue of T cells deficient in either caspase-8 or FADD can be achieved by simultaneously deleting RIPK1^156^ or by inhibiting RIPK1 pharmacologically with necrostatin-1^108^; furthermore, *FADD*^*−/−*^*RIPK1*^*−/−*^ and *Casp8*^*−/−*^*RIPK3*^*−/−*^ T cells undergo normal rates of clonal expansion following viral stimulation.^97, 156, 157^ Stimulation of the T-cell receptor by antigens induces the formation of the CARMA1-BCL-10-MALT1 complex which activates NF-*κ*B.^158, 159, 160, 161^ Downstream of this, a complex composed of FADD, caspase-8 and FLIP_L_ forms, which presumably prevents aberrant activation of RIPK1, blocking necroptosis and promoting cell survival and proliferation.^162^ TIPE2 (tumor necrosis factor-*α*-induced protein-8, TNFAIP8) is the newest member of the DED-containing protein family, identified as highly expressed in a murine model of spine inflammation.^163, 164, 165^ *TIPE2*^*−/−*^ mice develop spontaneous fatal inflammatory disease with concomitant elevated production of pro-inflammatory cytokines, suggesting a role for TIPE2 in the immune system and, more specifically, T cells.^164^ Upon infection or immunisation, *TIPE2*^*−/−*^ mice exhibit increased levels of CD8+ T cells and inflammatory cytokine production, implying that TIPE2 is a negative regulator of T-cell-mediated immunity by impeding the NF-*κ*B and AP1 transcriptional pathways and TLR signalling in macrophages.^164^ Interestingly, Sun *et al.*^165^ also found that TIPE2 interacts with caspase-8, but not FLIP, in macrophages, and blockade of caspase-8 function in *TIPE2*^*−/−*^ cells rescues the hypersensitive phenotype; however, subsequent papers have disputed this.

## Therapeutically Exploiting DED Complexes

Evasion of apoptosis is a hallmark of cancer,^166^ but aberrant cell death is also a feature of other human pathologies such as inflammation and neurodegenerative diseases. The DED family of proteins constitute key decision makers in these processes, with the ability to switch outcomes from life to death, or to different modes of death. As such, they represent an attractive set of proteins to target therapeutically.

Death receptors such as TRAIL-R1 and TRAIL-R2 are overexpressed in many types of cancer,^167^ and there has been much effort to develop agents (recombinant forms of TRAIL and antibodies) that activate these receptors, particularly as they appear to selectively target malignant tissue while sparing normal cells.^168^ Although pre-clinical data for TRAIL receptor-targeted therapeutics were promising and these agents were well tolerated in phase I trials, they showed limited anti-cancer effects in patients when used alone or in combination with chemotherapy or proteasome inhibition (reviewed in Lemke *et al.*^169^). However, a major shortcoming of these clinical studies was that they failed to learn from the experiences with other molecularly targeted agents and were conducted in unselected patient populations.^170, 171^ Another limitation of first generation TRAIL receptor agonists may have been insufficient levels of receptor super-clustering; a number of second generation TRAIL agonists are now in development with novel mechanisms of action that overcome this limitation, for example, MedImmune's multivalent ‘superagonist', which efficiently engages and clusters TRAIL-R2.^172^ However, increased valency may increase the toxicity of second generation TRAIL-R agonists, and a recent phase I clinical trial with a tetravalent agonistic Nanobody targeting TRAIL-R2, TAS266, had to be halted at the lowest dose owing to hepatotoxicity. ^173^ The opposite approach is required for the TNF*α* pathway, where biologics have been developed to block TNF*α* itself or TNFR1, preventing downstream activation of the NF-*κ*B pathway and/or apoptosis induction and providing effective treatment for a number of inflammatory diseases, including SLE, rheumatoid arthritis and septic shock.^174, 175, 176^ TNF*α*-induced necroptosis has been implicated in a number of pathophysiological conditions such as Crohn's disease^99^ (reviewed by Linkermann and Green^177^); thus, TNF*α* blockade may prove to be therapeutically beneficial in these situations.

A number of IAP antagonists (SMAC mimetics) are currently in clinical development and have shown potential as anti-cancer agents (reviewed by Fulda^178^). They promote caspase activation and elicit an apoptotic response by binding to and inhibiting IAPs, which are overexpressed in many types of cancer.^70, 71, 179, 180^ Additionally, they activate the non-canonical NF-*κ*B pathway through the accumulation of NIK, which is normally degraded by cIAP1.^181, 182^ This results in an upregulation of NF-*κ*B target genes, including TNF*α*. TNF*α* can signal in an autocrine or paracrine manner, stimulating the assembly of TNFR1 Complex II (Figure 6) and activation of cell death via apoptosis and/or necroptosis.^86, 92^

As it is a potent anti-apoptotic molecule, lowering FLIP expression in malignancies could lower the threshold for cell death. We and others have reported that histone deacetylase inhibitors such as vorinostat trigger the rapid ubiquitination and degradation of FLIP, thus sensitising cells to TRAIL or chemotherapy.^122, 170^ Additionally, a number of chemotherapeutics and other anti-cancer agents downregulate FLIP expression via multiple mechanisms (reviewed by Safa^183^). As previously mentioned, the gene encoding procaspase-8 (*Casp8*) is silenced by methylation in several cancers, such as small cell lung cancer and neuroblastomas;^184^ treatment with another class of epigenetic drugs, the demethylating agents such as 5-azacytidine can reverse this effect, thereby enhancing the potential for caspase-8-mediated apoptosis.^27^

## Conclusion and Perspective

As a result of their key roles in determining life and death outcomes, much work has focussed on the complexes formed by DED proteins. From this work, FLIP in particular has emerged as a master regulator of the signalling outputs from DED-containing complexes. It is probably for this reason that FLIP expression is regulated at multiple levels: by numerous transcription factors (such as NF-κB,^185^ NFAT,^186^ AP-1^187^ and c-Myc^188^); alternative splicing;^189^ mRNA translation^190^ and by posttranslational modifications, including its rapid turnover via the ubiquitin-proteasome system.^118, 119^ This exquisite level of regulation may have evolved to allow swift responses to various cellular stresses, for example, to safeguard against inappropriate activation of cell death or enhance cell death, depending on the cellular context. Biochemical and structural studies have demonstrated that DED-containing complexes are highly intricate with ubiqutination playing a key role. These complexes are also more numerous than previously appreciated, with the discovery of complexes such as the intracellular DISC and Ripoptosome and involvement of DED proteins in complexes such as the inflammasome. It is anticipated that future studies will reveal novel ways of therapeutically targeting DED protein complexes that could find clinical applications in cancers, inflammatory diseases and neurodegenerative diseases.

## Abbreviations

AIM2: absent in melanoma 2
AP-1: activator protein 1
ASC: apoptosis-associated speck-like protein containing a CARD
BCL-10: B-cell lymphoma/leukemia 10
BCL-2: B-cell lymphoma 2
BCLAF1: BCL-2-associated transcription factor 1
CARD: caspase activation and recruitment domain
CARMA1: CARD-containing MAGUK protein 1
CD95: cluster of differentiation 95
cIAP: cellular inhibitor of apoptosis
DD: death domain
DED: death effector domain
DEDD: death effector domain-containing protein
DISC: death-inducing signaling complex
FADD: Fas-associated protein with death domain
FLIP: FLICE-like inhibitory protein
HOIL-1: haem-oxidized IRP2 ubiquitin ligase 1
JNK: c-Jun N-terminal kinase
LGP2: Laboratory of Genetics and Physiology 2
MALT1: mucosa-associated lymphoid tissue lymphoma translocation protein 1
MAVS: mitochondrial antiviral-signaling protein
MCL-1: myeloid cell leukemia 1
MDA5: melanoma differentiation-associated protein 5
MLKL: mixed lineage kinase-like domain
NEMO: NF-κB essential modulator
NFAT: nuclear factor of activated T cells
NLR: NOD-like receptor
NLRP3: NOD-like receptor family, pyrin domain containing 3
PEA-15: phosphoprotein enriched in astrocytes 15
PIDD: p53-induced death domain
PTEN: phosphatase and tensin homolog
RIG-1: retinoic acid-inducible gene 1
RIPK: receptor-interacting serine/threonine protein kinase
SLE: systemic lupus erythematosus
SMAC: second mitochondria-derived activator of caspases
TAK1: transforming growth factor beta-activated kinase 1
TIPE2: tumor necrosis factor-*α*-induced protein 8 (TNFAIP8)-like 2
TRADD: TNF receptor type 1-associated death domain
TRAF: TNF-receptor associated factor
